# Myeloperoxidase and troponin T are linked with myocardial infarction among young Indians

**DOI:** 10.6026/97320630018066

**Published:** 2022-01-31

**Authors:** Anoop Jaiswal, Beenu Doctor, Manish Kumar Verma, Amrita Vamne

**Affiliations:** 1 Department of Biochemistry Index Medical College, Malwanchal University Indore, MP, India; 2 Department of Biochemistry, Moti Lal Nehru Medical College, Prayagraj, UP, India; 3 Department of Biochemistry, GSVM Medical College, Kanpur, UP, India

**Keywords:** Myocardial infarction, myeloperoxidase, Troponin T, Sandwich ELISA

## Abstract

It is of interest to document the correlation of myeloperoxidase (MPO) and Troponin T (TnT) in young Indian patients with myocardial infarction using epidemiological data. A study with 220 (110 case and 110 control) participants with Acute Coronary Syndrome (both males and females) was completed. Patients were identified using a pre-designed PROFORMA at the OPD and IPD of the Medicine Department at the Index Medical College & Hospital, Malwanchal University and Moti Lal Nehru Medical College and Swaroop Rani Nehru Hospital in India. MPO and TnT were measured using the Sandwich ELISA (Sandwich Enzyme linked immunosorbent Assay) kit. TnT (pg/ml) (Mean ± SD) were high in the case group than control 134.7 ± 8.57 vs 96.6 ± 2.82; F = 145.9; t = 44.3; p < 0.01). Moreover, MPO was 3-fold high in case group than control (157±30.1 and 42.4±14.82, respectively). The differences was found statistically significant (p<0.001). This suggests a link between TnT and MPO among young Indian patients with myocardial infarction.

## Background:

Myocardial infarction (MI) continues to be a significant problem in developed countries and is becoming an increasingly significant problem in developing countries [1]. Indians develop coronary artery disease (CAD) 5 to 10 years earlier than in other populations and the occurrence of first myocardial infarction before the age of 40 years is 5 to 10 folds higher [2]. Myocardial infarction (MI) is the most dangerous form of CHD (coronary heart disease), and it can cause immediate death. Although MI is more common in people over the age of 45, it can also affect young men and women. Fortunately, it does not affect people under the age of 45 years [3]. When the disease strikes at an early age, however, it causes severe morbidity and psychological impacts [4]. Because of atypical presentation and reluctance to submit to further investigations, the figures for young patients may be lower than actual. ICHD among those under the age of 40, on the other hand, was shown to account for only 3% of all CHD cases [5]. Risk factors are becoming more prevalent in young adults and youngsters. In the near future, this will lead to a rise in illness burden [6]. There are also increased levels of MPO and Troponin - T in patients with CHD [7]. Myocyte necrosis results from thrombus development at a ruptured or degraded plaque and distant embolization of platelet aggregates [8]. Assessing the release of troponins (Tns) have emerged as an important tool for risk assessment and therapeutic management of patients with ACS (Acute Coronary Syndrome) [9,10]. Myeloperoxidase is a member of the heme peroxidase super family and is found in the granules of Azurophilic leukocytes that are excreted during leukocyte activation [11]. The serum level of myeloperoxidase is known to be useful in predicting short and long-term prognosis following ACS, MI and cardiac failure [12- 15]. Polymorphisms in the myeloperoxidase gene have been linked to premature ischemic heart disease [16], ischemic heart disease intensity [17], and heart failure mortality [18]. The diagnostic and prognostic impairment of myeloperoxidase serum level has also been documented [19-20]. Therefore, it is of interest to document the correlation between myeloperoxidase and troponin T in young Indian patients with myocardial infarction.

## Materials and methods:

A study with 220 (110 case and 110 control) participants with Acute Coronary Syndrome (both males and females) was completed. Patients were identified using a pre-designed PROFORMA at the OPD and IPD of the Medicine Department at the Index Medical College & Hospital, Malwanchal University and Moti Lal Nehru Medical College and Swaroop Rani Nehru Hospital in India.

## Inclusion criteria:

1) Patients more than 18 years of age with ECG findings and biochemical markers.

2) Suggestive of acute myocardial infarction.

3) Elevated value of CK-MB and TnT.

4) Chest pain lasting 24 hours, suggestive of myocardial ischemia of accelerated pattern, or a prolonged one (> 20 minutes), or with recurrent episodes at rest, or at minimal exertion.

## Exclusion criteria:

1) Known causes of elevated uric acid level (chronic kidney disease, gout, hematological malignancy and hypothyroidism).

2) Patients on drugs which increase serum uric acid, for example, salicylates (2gm/dl, hydrochlorothiazide, pyrazinamide).

3) Chronic alcoholics.

4) Acute phase of impaired subject of obesity (body mass index > 30) will be excluded. In addition, patients receiving medications affecting lipid metabolism, such as lipid lowering drugs, beta-blockers, oral contraceptives, estrogen, progestin, thyroxin and vitamin E will be also excluded.

5) Present or past aspirin, statins or hormone replacement therapy, autoimmune diseases and malignancies smokers, subjects with any chronic diseases or acute infections, antioxidant vitamin supplements, hepatic disease etc.

## Sample collection:

The fasting blood samples were collected and centrifuged at 4000 RPM for 5 minute and stored at -80°C in a deep freezer.

## Estimation of MPO and TnT:

MPO and TnT were measured using the Sandwich ELISA (SandwichEnzyme linked immune sorbent Assay) kit method.

## Statistical Analysis:

Continuous data was analyzed using the Kolmogorov Smirov test. Non-normal continuous data was analyzed using the χ2 test. Mann Whitney U test were used to compare the groups. Categorical data were presented in frequency and percentage. Univariate binary logistic regression analysis was used to calculate unadjusted odds ratio (OR) and 95% confidence interval (CI) in different variables between case control for data on age. Baseline and principal data were compared between case and controls using 2 x 2 contingency table calculator available online at (http://faculty.vassar. edu/lowry/VassarStats.html). Statistical analysis was carried out using the statistical package SPSS-22, IBM, Chicago, USA. Two tailed p value < 0.05 has been considered as significant.

## Results:

### Baseline and principal characteristics of cases and controls:

The baseline characteristics of selected case and control groups are summarized in Table 1(see PDF). The mean age (± SD) of cases and controls are 44 ± 4.9 years and 45.32 ± 4.16years. Comparing the mean age of the two groups, Univariate binary logistic regression analysis showed similar (p>0.05) age between the two groups. In other words, subjects of two groups were age data matched. Further, gender data was also found similar (p>0.05) between the two groups. The principal characteristics are shown in Table 2(see PDF). Tobacco consumption was found to be a predominant risk factor for case group compared to control groups (OR: 182.3; 95% CI: 52-638; p: 0.001. Moreover, 2x2 chi square test showed that high level of smoking and use of alcohol were strongly associated with case risk (OR: 76.4; 95% CI: 22.6-257 and OR: 86.9; 95% CI: 25.7-294; all p<0.001). We also analyzed diabetes, STEMI and NSTEMI in case and control group. Ee enrolled healthy voluntary persons who came along with patients without these symptoms. Therefore, statistical comparison of these parameters between case and control group was statistically restricted.

### Distribution of gender characteristics on the basis of principal characteristics of cases and controls:

**Figure 1:**
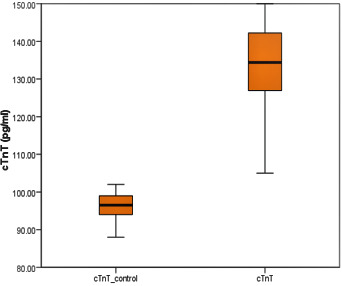
Box plot indicating the variation of TnT in case and control group. Line and asterisks indicate statistically significant difference, ***p<0.001). F=145.9; t=44.3; p<0.01

**Figure 2:**
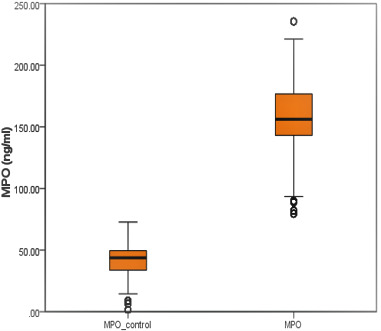
Diagram indicating the variation of MPO in case and control group. Line and asterisks indicate statistically significant difference, ***p<0.001). F=31.4; t=35.9; p<0.01

In this distribution case and control are subcategorized into male and female which were shown in Table 3(see PDF). Results revealed that in subgroup analysis of case having male verses female was not found to be null in selected parameters (all p>0.05). This result was similar in control group (all p > 0.05). In other words, subjects of two groups are same when compared between male and female in specific group. Significance was calculated between case and control using unpaired T test and a different scenario observed. Thus, all the selected biomarkers were found significantly high in case than its control group (p <0.05). TnT (pg/ml) (mean ± SD) were high in case group than control 134.7 ± 8.57 vs 96.6 ± 2.82; F=145.9; t=44.3; p < 0.01) (Figure 1). Moreover, MPO level was 3-fold high in case to control (157±30.1 and 42.4 ± 14.82, respectively) and the differences was found statistically significant (p < 0.001) (Figure 2). The comparisons concluded that TnT and MPO may have an influence on cases.

### Correlation between selected biochemical markers:

A positive but weak correlation was found for TnT with MPO. The relation of these selected markers was shown in Table 5 as shown in Figure 1 and Figure 2.

## Discussion:

MPO level was 3-fold high in Case to control (42.4±14.82) and the differences was found statistically significant (p:<0.001). The comparisons concluded that MPO may have an influence on cases and MPO level higher in cases than controls. Several researches have previously looked into the utility of MPO in predicting long-term effects. Li et al. [20] recently published a study that looked at 176 patients who had undergone coronary angiography. The patients were placed into four groups based on their MPO level quartile. They discovered that: (1) the ACS rate (36.2%) in the fourth quartile group of MPO level was 6 times higher than that in the first quartile group of MPO level, P.01; (2) the ACS rate (36.2 %) in the fourth quartile group of MPO level was 6 times higher than that in the first quartile group of MPO level; and (3) the ACS rate (36.2%) in the first quartile group of (2) The Gensini score in the fourth quartile of MPO level was substantially greater than the first quartile (P.01). Cavusoglu et al. [21] have also looked at the long-term prognostic value of baseline MPO levels in a well-characterized cohort of 193 males with ACS. All patients were followed prospectively for mortality and MI, and follow-up data for all patients was available at 24 months. The MI-free survival at 24 months for the group with MPO values below cut-off was substantially poorer than for those with values above cutoff, when the median MPO value of the entire cohort of patients (20.34 ng/mL) was used as a predetermined cutoff. Mocatta et al. looked at the link between plasma MPO and clinical outcome following an AMI. They looked at 512 AMI patients at hospital admission and tested plasma MPO in them, finding a significant link between MPO and follow-up events [22,23]. Omranet al. 2018 shows in a study that MPO was the most effective marker for diagnosing AMI in ACS patients when compared to other markers [24].

Hai et al. (2017) found that TnT (pg/ml) were high in case group than control (p<0.01), their study significant association was found in patient's groups than controls groups. They observe significant correlation in troponin level and the presence of atherosclerotic damage to the coronary arteries [25]. TnT provides for the detection of myocardial infarction at a later stage. Until 6 days following the start of infarct-related symptoms, the diagnostic efficiency continues at 98 percent [25]. According to Sikri et al. (2007) TnT can be used to track the success of thrombolytic therapy in individuals with myocardial infarction. TnT measures are very sensitive and specific for the early and late identification of myocardial injury, and hence could be used as a novel criterion in the laboratory diagnosis of myocardial damage [26].

Several investigations have revealed a link between MPO levels and coronary artery disease [27]. Elevated MPO levels indicated patients with undetectable troponin T (TnT) levels who were at increased risk of MI during their hospital stay or after discharge in a prospective trial of patients coming to the emergency department with chest pain [28]. MPO levels were found to be evenly distributed among individuals with low and high TnT serum levels, indicating that elevated MPO serum levels are not connected to myocardial damage in a time-dependent manner. Moreover, MPO identified patients at risk for cardiovascular events who had low baseline TnT serum levels. These data suggest that MPO is released prior to myocardial infarction and that MPO increase can detect patients with unstable atherosclerotic plaque formation even before micro vascular blockage [29]. In a recent study, MPO was revealed to be a robust predictor of poor outcomes in individuals with ACS people with low TnT levels were found as being at a higher risk for future cardiovascular events by MPO. This suggests that MPO conceals acute inflammatory conditions in the coronary circulation, which are linked to increased neutrophil activation and, as a result, myocardial damage [29]. We found a positive weak connection between TnT and MPO.

Data also showed that high level of tobacco smoking and use of alcohol was strongly associated with case risk in a study by Rosoff DB et al. (2020) alcohol consumption was also linked to an increased risk of myocardial infarction (MI) and coronary heart disease (CHD) [30] prior observational studies suggesting a link between drinking, smoking, and cardiovascular disease [31-32]. Notably, alcohol was not linked to lower CHD risk, which has been reported in observational studies to be the main CVD for which light-to-moderate alcohol consumption may be cardio protective [33], implying that previously reported reductions in CVD risk were due to other lifestyle differences associated with more light-to-moderate drinking patterns, such as healthier lifestyles rather than alcohol [34].

## Conclusion:

A significance association of myeloperoxidase and troponin T levels with MI among young Indians was observed. The elevated level of myocardial infarction contributes to ongoing myocardial damage. Myeloperoxidase and troponin T assay is a useful diagnostic test that can be performed to rule out myocardial infarction in patients with chest pain. Thus, recurrent sampling becomes critical for confirming a diagnosis in early presentations. Early cardiac biomarkers such as MPO in these conditions aid decision-making by boosting diagnostic effectiveness of MI for early treatment to reduce death.
